# Royal Medical and Chirurgical Society

**Published:** 1897-05-15

**Authors:** 


### Medical Progress and Hospital Clinics.

[*The Editor will be glad to receive offers of co-operation and contributions from members of the profession. All letters should be addressed to* The Editor, at the Office, 28 & 29, Southampton Street, Strand, London, W.C.]

### ROYAL MEDICAL AND CHIRURGICAL SOCIETY.

At the meeting held on April 27th, a paper was read by Dr. Francis Warner on the Relations between Bodily Development, Nutrition, and Brain Conditions in their Pathological Aspects, the object of the author being to show, as the result of extended observation among children, that—(1) Points of abnormal development in the body are very common, and have important signification; (2) that abnormal nerve-signs are much associated with defective development, as well as with the causes of mental dulness, and are of clinical importance; and (3) that there is a close empirical relation between (*a*) defective development of the body, (*b*) abnormal nerve-signs, (*c*) low nutrition of the body and its tissues, and (*d*) mental dulness and backwardness.

Some explanations of the relations among the various classes of defect might be offered. After referring to his paper on "Postures of the Hand as Indications of the Condition of the Brain," read before the Society in 1882, the author said that he had been enabled to examine 100,000 children, between 1888-94, individually in conjunction with other medical men, the results of which had been published. Notes had been taken of 18,127 cases in some respect below normal, and these formed a basis of facts which were presented in a series of tables. After briefly describing the method of statistical examination of facts, the author passed on to describe four main classes of defect observed: (*a*) Defect in development in size, form, proportion of parts of the body, principally in cranium, palate, ears, and features, as well as more gross deformities, such signs being disproportions in ratios of growth. (*b*) Abnormal nerve-signs in balance and action or reaction, in movement and co-ordination, as seen in the face and eyes, the upper extremities, the hands, digits, and balance of the body. (*c*) Low nutrition; cases pale, thin, or delicate. (*d*) Cases dull or backward mentally, as reported by the teachers.

The developmental signs were disproportions in growth; the nerve signs were abnormalities in the time of action, or in the ratios of action among the nerve-centres. The inference from the observed co-relation of development defect with abnormal nerve-signs was that when proportional growth in body is abnormal, as a sequence of inheritance or otherwise, the nerve-centres are commonly so built up that in subsequent action they do not work in the normal relations of time and quantity under the influence of the environment.

It was shown that disproportion in growth and development of the body is commonly accompanied by a condition of tissue prone to atrophy under adverse circumstances. This was specially the case with girls, who might therefore fall into permanent ill-health accompanied with neurosis. Inco-ordinated nerve action, such as fidgetiness, awkward habits, or chorea, commonly occurred with a lower status of nutrition.

The author proceeded to afford evidence upon the following propositions: (1) Disproportioned bodily development is very common; it is co-related with low nutrition of body, and inco-ordinated brain action. (2) Inco-ordinated brain action is much associated with mal-development, and the presence of nerve-signs is more co-related with the causes of mental dulness than are the signs of defective development. (3) Delicate children are in a large proportion of cases ill-developed in some part of the body. Among children without developmental defect there does not seem to be a larger number of delicate girls than boys; it is the girl who has some defect that is delicate, not all girls. Developmental cases tend to remain thin and delicate in all social classes, least so in resident institutions.

